# Dynamics and restriction of murine leukemia virus cores in mitotic and interphase cells

**DOI:** 10.1186/s12977-015-0220-2

**Published:** 2015-11-14

**Authors:** Efrat Elis, Marcelo Ehrlich, Eran Bacharach

**Affiliations:** Department of Cell Research and Immunology, The George S. Wise Faculty of Life Sciences, Tel Aviv University, Tel Aviv, Israel

## Abstract

**Background:**

Murine leukemia viruses (MLVs) naturally infect unsynchronized T and B lymphocytes, thus, the incoming virus encounters both interphase and mitotic cells. While it is well accepted that MLV requires cell division to complete its replication cycle, it is not known if ab initio infection of mitotic cells can result in productive infection. This question is highly relevant since the milieu of mitotic cells is markedly different from this of interphase cells; e.g. lacking radial microtubule network and intact nuclear envelope. To follow MLV infection in mitotic and interphase cells in real-time, we employed our recently developed infectious MLV particles with labeled cores, cellular models expressing fluorescence markers of different intracellular compartments and protocols for reversible mitotic arrest of MLV-susceptible cells.

**Results:**

Multi-wavelength live cell imaging was employed to simultaneously visualize GFP-labeled MLV cores, DiD-labeled viral or cellular membranes, and fluorescently-labeled microtubules or chromosomes. Cells were imaged either at interphase or upon mitotic arrest with microtubule poisons. Analysis of virus localization and trajectories revealed entry by endocytosis at interphase and mitosis, and correlation between viral mobility parameters and presence or absence of polymerized interphase microtubules. The success of infection of viruses that entered cells in mitosis was evidenced by their ability to reverse transcribe, their targeting to condensed chromosomes in the absence of radial microtubule network, and gene expression upon exit from mitosis. Comparison of infection by N, B or NB -tropic viruses in interphase and mitotic human cells revealed reduced restriction of the N-tropic virus, for infection initiated in mitosis.

**Conclusions:**

The milieu of the mitotic cells supports all necessary requirements for early stages of MLV infection. Such milieu is suboptimal for restriction of N-tropic viruses, most likely by TRIM5α.

**Electronic supplementary material:**

The online version of this article (doi:10.1186/s12977-015-0220-2) contains supplementary material, which is available to authorized users.

## Background

After entry into the cytoplasm of the infected cell, the retroviral core that harbors the reverse-transcribed DNA genome has to reach the chromosomes in order for integration to occur. The interactions of the core with cellular components along this route are not fully known. Microtubule-directed movements toward the nucleus were documented for HIV-1 cores [1, 2] and the involvement of the kinesin-1 adaptor protein—FEZ1—in this process has recently been demonstrated [3]. In addition, dynein and kinesin motors were implicated in the enhancement of HIV uncoating along these movements [4]. The importance of the microtubule network for viral trafficking and retroviral infection is further apparent by the HIV-induced formation of stable microtubules that enhances infection [5].

After traversing the cytoplasm, HIV-1 cores are thought to enter the nucleus through their interaction with nuclear pore proteins [6–11]. Unlike HIV-1, the murine leukemia virus (MLV) shows high tropism for dividing cells [12, 13] and its infection is thought to be dependent on the nuclear envelope (NE) breakdown during mitosis [12, 14]. Indeed, our previous microscopic analyses demonstrated that immediately upon the start of NE breakdown, MLV cores enter the nucleus and dock onto mitotic chromosomes [15]. In addition, exit from mitosis is required for integration of this virus [14]. Taken together, these requirements establish the need for passage through cell-cycle for MLV productive infection.

MLVs naturally infect T and B lymphocytes [16, 17]. Considerable portion of such lymphocytes—freshly isolated from lymph nodes of neonatal or adult mice—are cycling (~4–7 % for CD4^+^ cells and ~13–15 % for B220^+^ cells; [18]). This raises the question if this subpopulation of cells is equally susceptible to infection as interphase cells. This question is particularly relevant as the cellular milieu of mitotic cells is substantially different from this of interphase cells. Specifically, mitosis induces structural and functional alterations to the endocytic machinery, radial microtubule network, the presence or absence of intact NE and chromatin organization (reviewed in [19–21]), all potentially relevant to early and late stages of MLV infection. Moreover, cellular restriction factors that restrict HIV infection were shown to interact with and to be dependent on subset of these cellular features [22, 23]. Yet, most MLV infections were tested in unsynchronized cells (i.e. mainly interphase cells) and even in synchronized cells, the steps of MLV infection were not evaluated in the context of mitotic cells.

Here we used a p12-based system to label MLV cores for their detection at early steps of infection in interphase and mitotic cells. This system is based on the generation of MLV particles harboring cores that only portion of their p12 molecules are labeled with GFP. This results in labeled cores, which retain their infectious potential [15]. Using this system, we show that the mitotic cellular context affects the dynamics and restriction of MLV cores.

## Results

### MLV enters through the endocytic pathway in both interphase and mitotic cells

**Fig. 1:**
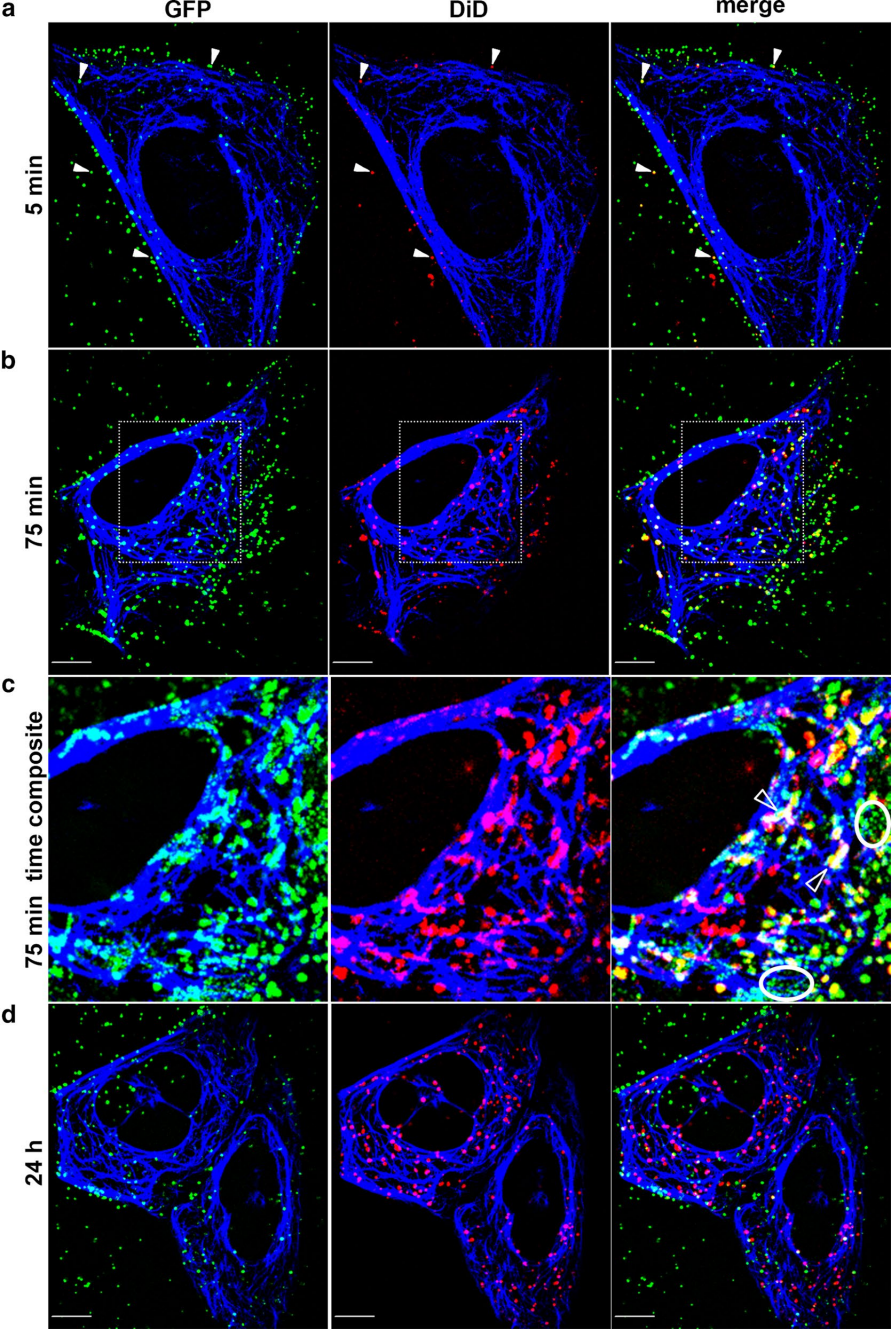
Entry and intracellular trafficking of MLV cores in interphase cells. Unsynchronized U/R/EMTB-mCherry cells were infected with DiD-labeled wt GFP virions and interphase cells were imaged at 5 min (**a**), 75 min (**b**, **c**) and 24 h (**d**) postinfection. All *panels* are single optical slices obtained by spinning-disc confocal microscopy. *Panel* **c** shows magnified time composites of insets in (**b**), prepared from Additional file 1: Mov. S1. Viral cores are in *green*; DiD signal and EMTB-marked microtubules are pseudocolored in *red* and *blue*, respectively. Full arrowheads mark GFP^+^DiD^+^ particles in a single confocal plane. In time composite images, *empty* *arrowheads* mark paths of GFP^+^DiD^+^ particles overlapping microtubules; *ellipses* enclose paths of GFP^+^DiD^−^ cores presenting minimal displacement and not localized to microtubules. *Scale bars* 10 µm

Ecotropic MLV is thought to enter through the endocytic pathway that provides low pH and cathepsins, required for fusion between the viral and cellular membranes. Accordingly, the fusion step is pH-sensitive and thought to occur not at the plasma membrane but rather at the endosomes [24–29]. Whereas the clathrin-mediated endocytic route is constantly active in interphase cells [30], some reports suggested that this pathway may be selectively reduced in mitotic cells [19, 31, 32]. To study MLV entry in interphase and mitotic cells, we co-stained GFP-labeled MLV virions with DiD—a lipophilic dye [33–35]. The GFP-labeled virions (named ‘wt GFP’, [15]) consisted of MLV cores, co-assembled with GFP-p12 fusion molecules; and their labeling with DiD was achieved by incubation of 293T cells, transfected with plasmids expressing the components of wt GFP with 5 µM DiD. Thus, GFP and DiD label different viral components, i.e. GFP the MLV cores and DiD the virion membrane. Spotting aliquots of the culture supernatant on glass coverslips and imaging by fluorescence microscopy revealed that 50–60 % of GFP-labeled virions showed detectable DiD fluorescence. Yet, under live-cell imaging conditions (shorter exposures) only approximately one-third of these particles were detected as doubly-labeled (GFP^+^DiD^+^; Fig. 1a). This is in line with the partial incorporation of the dye into retroviral particles, detected in earlier studies [33, 34]. Few DiD-only signals (which did not co-localize with GFP-marked cores) were also observed; likely representing membranous vesicles, originating from the donor cells. In additional experiments (n = 3), wt GFP virions—harboring a MLV-based vector (pQCXIP-GFP-C1) that expresses the EGFP marker (see below and in [36])—were labeled, or not, with DiD and the infectivity of these particles was quantified (Methods). Labeled particles yielded 1.3 ± 0.3 fold infectivity higher than unlabeled particles (n = 3). This analysis demonstrated that DiD labeling did not reduce wt GFP infectivity.

Virion-containing supernatants were incubated with unsynchronized or nocodazole-arrested U/R/EMTB-mCherry cells. These cells originated from U/R cells (U2OS human osteosarcoma cells that express the MLV receptor [37]), which also express the EMTB-mCherry fusion protein (composed of two mCherry repeats fused to the N-terminus of the microtubule binding-domain of ensconsin; EMTB [38]). The infected cultures were visualized by live-cell imaging during the first 2 h of infection, after which the cells were washed and re-visualized 24 h postinfection. The labeling of different viral components (membrane vs. cores) allows for analysis of entry. Specifically, the double-labeling (GFP^+^DiD^+^) is indicative of enveloped particles before fusion. Conversely, singly-labeled particles (GFP^+^DiD^−^) represent two classes, either particles that were not labeled with DiD or post-fusion cores. In interphase cells, as early as 5 min after exposure of the cells to the labeled virions, the majority of particles decorated the cell contour, suggesting plasma membrane localization, while minor portion of the labeled particles already localized to the cell interior. A measurable portion of membrane-attached particles were double-labeled (GFP^+^DiD^+^) (Fig. 1a; yellow dots) and the proportion of double labeled particles was similar for the particles attached to the glass or to the plasma membrane, suggesting that the DiD labeling does not affect cell attachment of particles.

At 75 min postinfection, the distribution and dynamics of labeled particles differed from those observed at 5 min. Namely, a greatly increased number of particles were intracellular (localizing to the area occupied by stained microtubules, pseudocolored in blue; compare Fig. 1b–a) and motile (Fig. 1c and Additional file 1: Mov. S1). Within the cell, the portion of GFP^+^DiD^+^ relative to all GFP-labeled puncta was 30 % (Fig. 1b, c). The notion that these double-labeled particles represent incoming virions engulfed in endocytic compartments is supported by their retention of DiD label (marking viral lipid envelopes) and the movement of subset of these particles along microtubules (marked with EMTB-mCherry; Fig. 1c, empty arrowheads and Additional file 1: Mov. S1 and Additional file 2: Mov. S2; and see below movement analyses). Such GFP^+^DiD^+^ particles presented saltatory movement, which was heterogeneous in terms of path length, velocity and confinement (Methods; Microscopy). Cytoplasmic GFP^+^ complexes that were not labeled with DiD (GFP^+^DiD^−^) were also observed (Fig. 1b, c); a portion of these likely represent MLV cores that were released from the endosomes after membrane fusion. The latter cores did not move along microtubules but rather showed undirected and limited displacement (Fig. 1c, ellipses), as we reported before [15].

Since GFP^+^DiD^+^ particles moved along microtubules, we proceeded to compare their movement parameters with those of endosomes in U2OS cells. To visualize the dynamics of these endosomes, we transfected U2OS cells with plasmid expressing the endosomal marker FYVE-GFP. Time-lapse sequences of the transfected cells (Additional file 3: Mov. S3) revealed two sub-populations of labeled puncta: small-motile and large puncta showing restricted motility (Additional file 4: Mov. S4), suggesting that different sub-classes of endosomes are found in U2OS cells. FYVE-GFP-labeled endosomes, like the GFP^+^DiD^+^ cores, presented maximal velocities in the range of 0.2–2 µm/s. These values fit those reported for transport of endosomes [39, 40] and other viral particles (discussed in [2]) along microtubules. This concordance of values further supports the notion that the movement of the GFP^+^DiD^+^ cores reflects their inclusion in endosomes. To directly test the localization of GFP^+^DiD^+^ particles to intracellular endocytic structures, we fed GFP^+^DiD^+^-infected U/R cells with fluorescently labeled transferrin, which is internalized by clathrin-mediated endocytosis and labels early and recycling endosomes. These cells were co-labeled with Hoechst 33342 dye (to visualize nuclei, shown in white) and imaged by spinning disk confocal live cell microscopy (Additional file 5: Fig. S1). Fluorescently-labeled particles showing signal in all three channels (blue- transferrin, red- viral membranes, and green- viral cores) were readily detected (Additional file 5: Fig. S1 and Additional file 6: Mov. S5). Moreover, the triple co-localization of signals persisted through multiple time points of the time lapse (Additional file 6: Mov. S5). Taken together, our data firmly demonstrate the endocytosis and endosomal localization of GFP^+^DiD^+^ particles.

At 24 h post infection, the near entirety of puncta was singly-labeled, either with GFP or DiD (Fig. 1d, Additional file 7: Fig. S2). Such scenario is in accord with post-fusion events, in which the GFP-labeled cores segregated from DiD-labeled cellular endocytic compartments. Moreover, green and red labeled puncta differed in size (average of 0.7 ± 0.04 and 0.4 ± 0.03 µm^2^ for red and green puncta, respectively) and motility (Additional file 7: Fig. S2; Additional file 8: Mov. S6). To quantify the difference in motility between the red and green puncta we visualized the “footprint” of each particle by adding its emitted signal overtime, i.e. time composite (Additional file 7: Fig. S2). We then calculated the difference in area of green and red signal between the initial frame and the time-composite over 2 min of a confocal time-lapse series (Additional file 8: Mov. S6). While the area occupied by the green signal increased 20 fold, in accord with motile particles; a much lesser increase (fourfold) was calculated for area of the red signal. Taken together, these data demonstrate that in interphase cells (ecotropic) MLV cores enter via endocytosis and are released from endosomes into the cytoplasm.

**Fig. 2:**
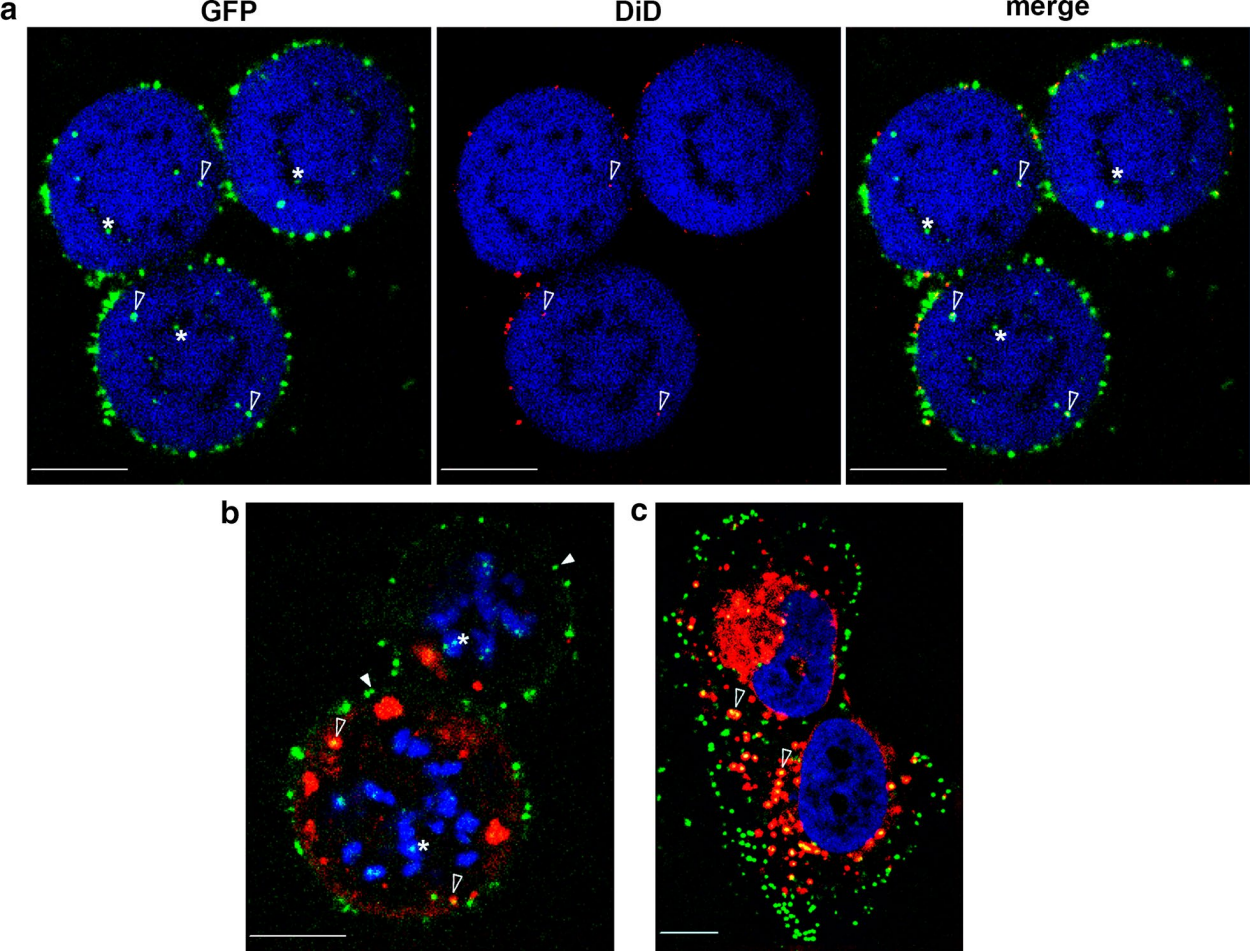
Imaging MLV Entry into mitotic cells. **a** U/R/EMTB-mCherry cells, arrested at mitosis by nocodazole, were infected with DiD-labeled wt GFP virus and visualized 75 min postinfection. Viral cores and viral membranes are in *green* and *red*, respectively. Cellular EMTB fluorescence is pseudocolored in *blue*. *Empty arrowheads mark* intracellular GFP^+^DiD^+^ puncta. *Asterisks* *mark* intracellular GFP^+^DiD^−^ puncta, overlapping dark regions, likely representing mitotic chromosomes. Nocodazole-arrested (**b**) or interphase (**c**) U/R/H2A-RFP cells, whose cellular memebranes were labeled with DiD (*red*), were infected with wt GFP virus (*green*) and visualized 1 h postinfection. *Full arrowheads mark* peripheral/extracellular cores that do not overlap the DiD staining. *Empty arrowheads mark* intracellular viral cores overlapping dense DiD staining. *Asterisks mark* viral cores attached to mitotic chromosomes, pseudocolored in *blue*. *Scale bars* 10 µm

In previous studies we imaged GFP-labeled cores in infected cells that progressed through cell cycle from interphase to mitosis [15]. The early phases of these infections (i.e. entry and endosomal escape) occurred in interphase cells. Yet, the susceptibility of cells undergoing mitosis to initial stages of MLV infection (ab initio infection) is unknown. To address this issue, we first arrested U/R/EMTB-mCherry cells in mitosis (prior to anaphase) with either 2-methoxyestradiol (2ME2) or nocodazole [15], and exposed these arrested cells to DiD-labeled wt GFP. Imaging of the cells revealed that whereas interphase cells were characterized by flat appearance and extensive radial microtubule array (Fig. 1), arrested cells showed rounded shape with an essentially diffuse signal of EMTB-mCherry (pseudocolored in blue; Fig. 2a). In these cells, condensed chromosomes appeared as dark, intracellular unstained regions. At 5 min post exposure to the virus, GFP^+^DiD^+^ and GFP^+^DiD^−^ particles were observed attached to the plasma membrane (data not shown), suggesting that MLV particles bind the membrane of mitotic cells. At 75 min postinfection, in addition to particles localized to the plasma membrane, cytoplasmic GFP^+^DiD^+^ particles were also observed, suggesting the use of the endocytic pathway for virus entry in mitotic cells (Fig. 2a; empty arrowheads). To compare the efficiency of viral entry in mitotic and interphase cells, we calculated the portion of intracellular GFP-labeled puncta (overlapping with the EMTB-mCherry signal in single confocal planes). Notably, both the overall number of GFP puncta (i.e. internalized puncta and puncta found on the external cell surface) and the portion of internalized GFP puncta were lower in mitotic cells. Specifically, when 18 interphase or mitotic cells were examined, an average of 290 ± 33 GFP puncta per interphase cell and 108 ± 8 GFP puncta per mitotic cell were observed; out of which 86 and 33 % were localized inside the cell in interphase and mitotic cells, respectively (equivalent to an average of ~70 and ~40 particles per cell at the plasma membrane in mitotic and interphase cells, respectively). This scenario is in accord with reduction in uptake of labeled MLV in mitotic cells. To clarify if the increase in number of plasma membrane-localized particles in mitotic cells resulted from differences in membrane localization of mCAT-1 MLV receptor, we stably expressed a fluorescently tagged mCAT-1 (mCAT-1–mStrawberry, [41]) in U20S cells. These cells were arrested with 2ME2, or not, and receptor localization was visualized by confocal microscopy. In both interphase and mitotic cells, mCAT-1–mStrawberry showed a prominent localization to the plasma membrane (Additional file 9: Fig. S3). To quantify the portion of receptors localized to the plasma membrane, we measured the mean intensity of fluorescence signal (reflective of receptor densities) at the plasma membrane and compared it to adjacent intracellular regions. With such protocol, we aimed at reducing the potential effect of differences in expression levels of mCAT-1–mStrawberry, by calculating a ‘per cell’ ratio. The calculated ratio revealed a slight but significant higher ratio in interphase cells (1.37 fold, p < 0.04), suggesting a minimal decrease in portion of receptors localizing to the plasma-membrane in mitosis. The inverse correlation between membrane localization of receptor (~1.4 higher in interphase cells) and membrane localization of virus (1.8 fold higher in mitotic cells) points to the internalization step as a major regulatory determinant of the lesser number of intracellular cores in mitotic cells. Such scenario is in accord with the differential regulation of endocytosis in mitotic cells [19, 31, 32].

When quantitative analysis was applied for comparison of movement of intracellular, wt GFP cores in mitotic and interphase cells, we observed a reduction in the values of the maximal velocities of the labeled particles in the mitotic cells (Additional file 10: Fig. S4A; a left-shifted distribution of frequencies for mitotic cells). Mean square displacement (MSD) analysis of the movement of multiple viral cores in interphase cells showed heterogeneity in the slope and curve shape (MSD versus time graphs; Additional file 10: Fig. S4B). Typically, curves could be grouped into two subpopulations, presenting either a linear correlation between MSD and time, indicative of unperturbed diffusion (graphs labeled in blue colors); and a group showing an exponential-like pattern (labeled in red colors), suggestive of active transport. Notably, upon similar analysis applied to mitotic cells (Additional file 10: Fig. S4C), no curves of the latter category were observed, in line with the absence of polymerized microtubules. In these mitotic cells, in addition to freely diffusing particles (linear MSD/time ratio; graphs labeled in blue colors) we observed particles presenting anomalous curves (labeled in gray), for which we do not yet have an interpretation. Importantly, in mitotic cells we could also detect GFP^+^DiD^−^ particles that overlapped dark areas inside the cells (Fig. 2a, asterisks), likely representing cores attached to mitotic chromosomes (see below). Taken together, these data reinforce the notion that the intracellular milieu of cells at different stages of the cell cycle influences the dynamics and motility of incoming viral cores.

Since ecotropic MLV requires the endosomal environment, which provides both low pH and cathepsins, for entry by fusion [24–29], we wanted to directly probe if wt GFP reaches internal membranous compartments in mitotic cells. To investigate this, we marked the lipid membranes of U/R/H2A-RFP cells (U/R cells that stably express the red fluorescent protein fused to histone H2A, which marks the chromosomes; [15] ) with DiD and washed away the unbound dye. Cells were incubated or not with nocodazole (24 h); treated and untreated cells were infected with wt GFP particles (for 1 h). In both nocodazole-treated and untreated cells prominent DiD staining on internal membranous compartments was observed (pseudocolored red; Fig. 2b, c). In single confocal mid-planes of mitotic cells, GFP signals (total of 150 dots, counted in eight cells) distributed into three categories: the first (55 % of total particles) consisted of peripheral (non-internalized) particles (Fig. 2b, full arrowheads); the second (30 %) overlapped with internal DiD-labeled membranes (Fig. 2b, empty arrowheads) and the third type (15 %) of GFP signal overlapped the chromosomal signal (pseudocolored blue, Fig. 2b, asterisks). The overlap between GFP and internal, DiD-labeled membranes implies for particles engulfed in endocytic compartments. This notion was further supported by the spatial restriction of the movement of the engulfed particles, which did not trespass the borders of the DiD-labeled endosomes (Additional file 11: Mov. S7). Such overlap between GFP and internal DiD-labeled membranes could be detected also in interphase cells (Fig. 2c, empty arrowheads). These results further suggest that the entry of ecotropic MLV to mitotic cells occurs by the endocytic pathway, similar to the entry into interphase cells. The overlap between GFP signal and the chromosomes suggests that MLV cores that entered mitotic cells could exit the endocytic compartments (see below).

### Viral cores target mitotic chromosomes in the absence of radial microtubule network

**Fig. 3:**
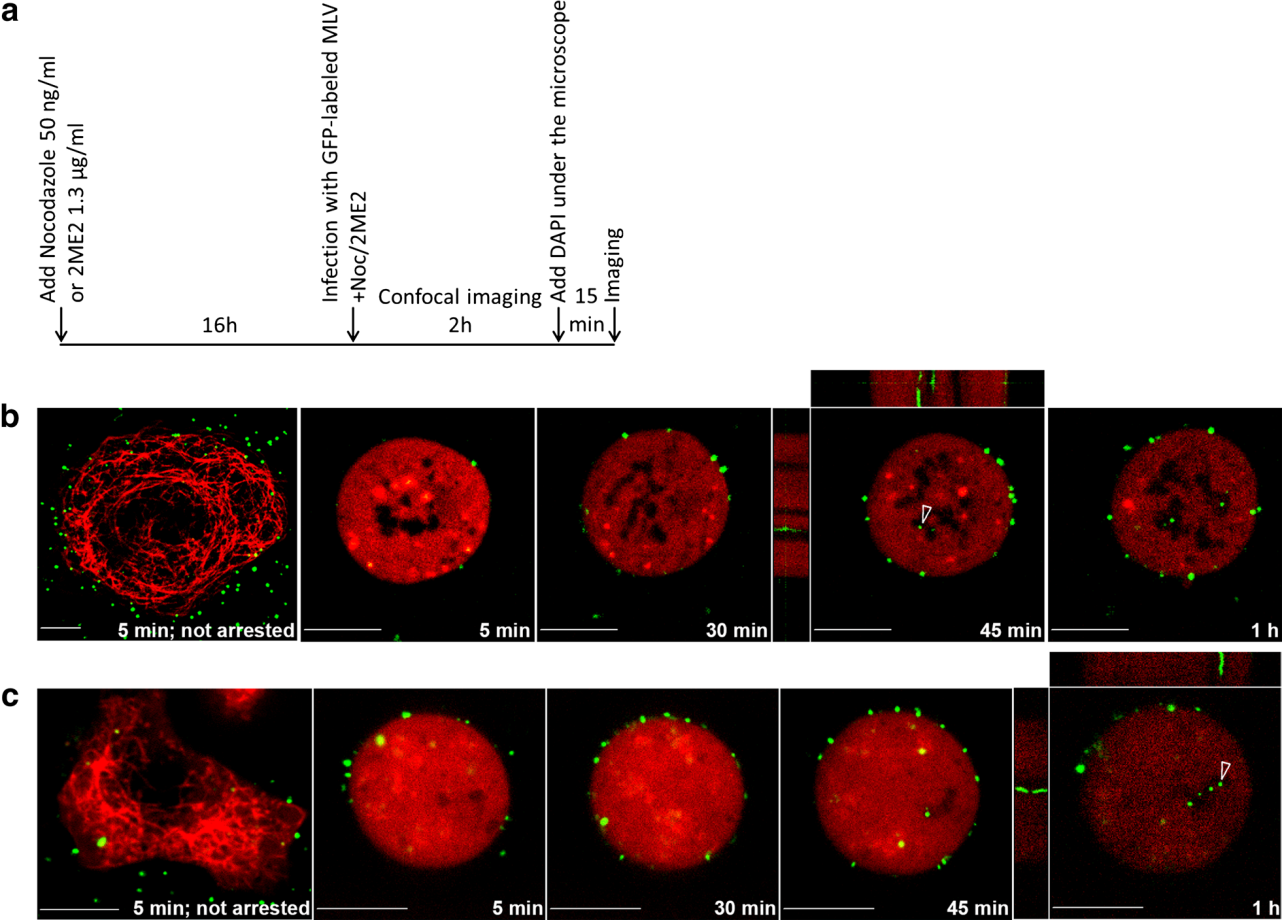
Viral cores reach and dock to mitotic chromosomes in absence of polymerized microtubules. **a** Timeline for infection during mitotic arrest. U/R (**b**) or NIH3T3 (**c**) cells expressing EMTB-mCherry (*red*) were treated with 2ME2 (**b**) or nocodazole (**c**), infected with wt GFP virions. The *left images* in (**b**) and (**c**) show drug-treated cells, not arrested at mitosis; while the other images in each *panel* show a representative arrested cell, imaged at the indicated times postinfection. GFP-labeled MLV cores are in *green* and condensed chromosomes appear as intracellular dark regions. Kymographs of selected time points [45 min (**b**; Additional file 12: Mov. S8) or 1 h (**c**)] show immobile MLV cores (appear as continuous *green lines* [15]), docked to mitotic chromosomes (examples are indicated by *empty arrowheads*). *Scale bars* 10 µm

The presence of labeled cores on condensed chromosomes (Fig. 2a, b) suggested that MLV cores are able to traffic from the plasma membrane to mitotic chromosomes in the absence of microtubule network. To further test this, we arrested, prior to infection, cells at mitosis. This was achieved using either nocodazole or 2ME2 (as in [15]) in U/R/EMTB-mCherry cells or NIH3T3 cells expressing this fluorescent EMTB (NIH3T3/EMTB-mCherry). Such cells were infected with wt GFP and cells lacking radial microtubules were imaged (timeline in Fig. 3a). Real-time imaging of an arrested cell (Fig. 3b) detected GFP-labeled particles at the plasma membrane as early as 5 min postinfection, and the number of membrane-bound particles increased over 30 min postinfection. At 45 min postinfection, clear docking of the labeled viral cores to the mitotic chromosomes (dark intracellular regions) was detected. A kymograph of time-lapse imaging of these cores for 23 s demonstrated their stable docking to the chromosomes (Fig. 3b and Additional file 12: Mov. S8). Additional docking events were observed at 1 h postinfection (Fig. 3b). Similar results were obtained using NIH3T3/EMTB-mCherry, treated with nocodazole (Fig. 3c). DAPI (4′,6-diamidino-2-phenylindole) staining of these cells further emphasized the docking of the GFP-labeled cores to mitotic chromosomes (Additional file 13: Mov. S9).

Overall, these results suggest that in mitotic cells, in the absence of a microtubule network, MLV cores traffic from the plasma membrane to the chromosomes, where they stably dock.

### Viral cores that target mitotic chromosomes in the absence of microtubule network support productive infection

**Fig. 4:**
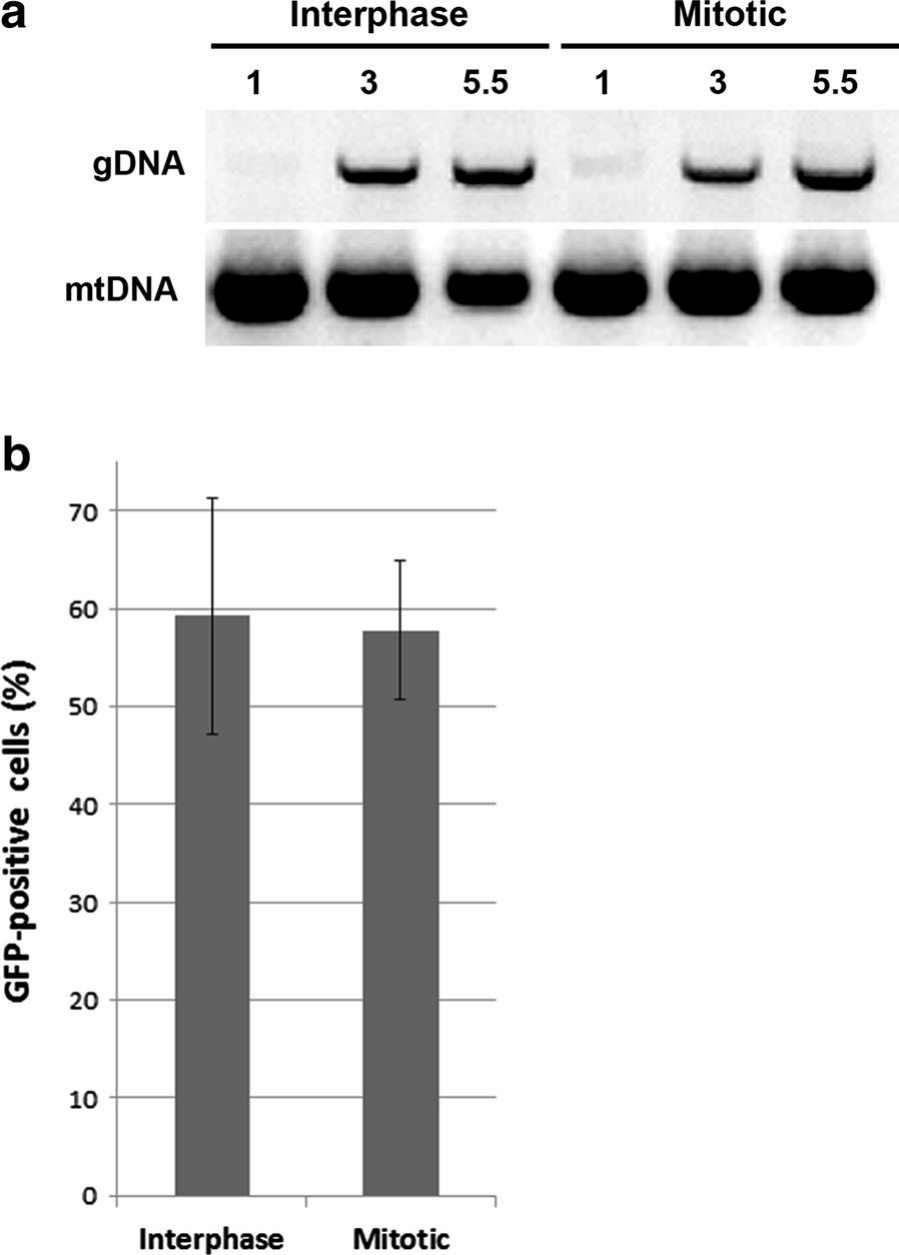
Reverse transcription and expression of MLV genome in cells infected in absence of microtubules. **a** Detection of reverse transcription products by PCR in interphase and mitotic cells. Low molecular DNA was extracted from interphase and mitotic cells at the indicated hours postinfection. Primers derived from matrix-capsid region or from cytochrome B gene were used to amplify gDNA or mtDNA, respectively. Shown are the PCR products, separated by gel electrophoresis. **b** Unsynchronized (Interphase) or nocodazole-arrested (Mitotic) cells were infected with MLV virions that co-packaged the pQCXIP-GFP-C1 vector. The cells were allowed to cycle, beginning at 6 h post infection, and 2 days later, the percentage of GFP-positive cells was determined by FACS. *Bars* represent the mean ± standard error of the means (n = 3)

To further characterize initial steps of MLV infection, we assessed reverse transcription in mitotic (nocodazole-arrested U/R cells) and interphase U/R cells, infected with wt MLV. Equal numbers of these cells were re-plated following their resuspension either by trypsinization (interphase cells) or the mitotic shakeoff method (mitotic cells; Methods). At 1, 3 and 5.5 h postinfection, Hirt extraction [42] was performed to obtain low molecular DNA from the cells and PCR was used to amplify genomic viral DNA (gDNA). Amplification of mitochondrial DNA (mtDNA) was used to control for the efficiency of the extraction. PCR reactions clearly revealed an increase in gDNA over time, both in interphase and mitotic cells (Fig. 4a), demonstrating that reverse transcription occurred in cells arrested at mitosis.

Having observed reverse transcription in mitotic cells, we next quantitatively measured the percentage of infected cells, expressing GFP marker that is derived from a retroviral vector. For this, we infected nocodazole-arrested U/R cells with wt MLV that co-packaged a MLV self-inactivated (SIN) vector, harboring EGFP and puromycin-resistance genes (pQCXIP-GFP-C1; [36]). In preliminary experiments, cells that were replated following shakeoff failed to efficiently reenter the cell cycle, a pre-condition for efficient integration [14]. Thus, we performed the experiment in attached cells, taking into account that our nocodazole-treatment (for 16 h) resulted in mitotic arrest of ~60 % of the treated cells [measured by Fluorescence-activated cell sorting (FACS), data not shown]. Arrested and non-arrested cultures were exposed to the virus for 6 h (in the presence or not of nocodazole, respectively), after which the supernatants were discarded; the cells were washed and incubated with complete medium for 2 days to allow the cells to exit mitosis. The percentage of GFP-positive cells was then determined by FACS analysis, as a measurement for the expression from the integrated vector. Of note the SIN vector cannot spread and thus such percentage reflects the initial portion of infected cells. This analysis revealed comparable percentages of GFP-positive cells for cultures that were either unsynchronized, or arrested at mitosis, at the time of infection (Fig. 4b). Since more than half of the cells in the arrested culture were in mitosis at time of infection, this implies that initial MLV infection in the absence of a polymerized microtubule network, results in integration and expression of the provirus. These integration and expression steps likely occurred upon exit from mitosis [14].

### TRIM5α restriction is reduced in mitotic cells

**Fig. 5:**
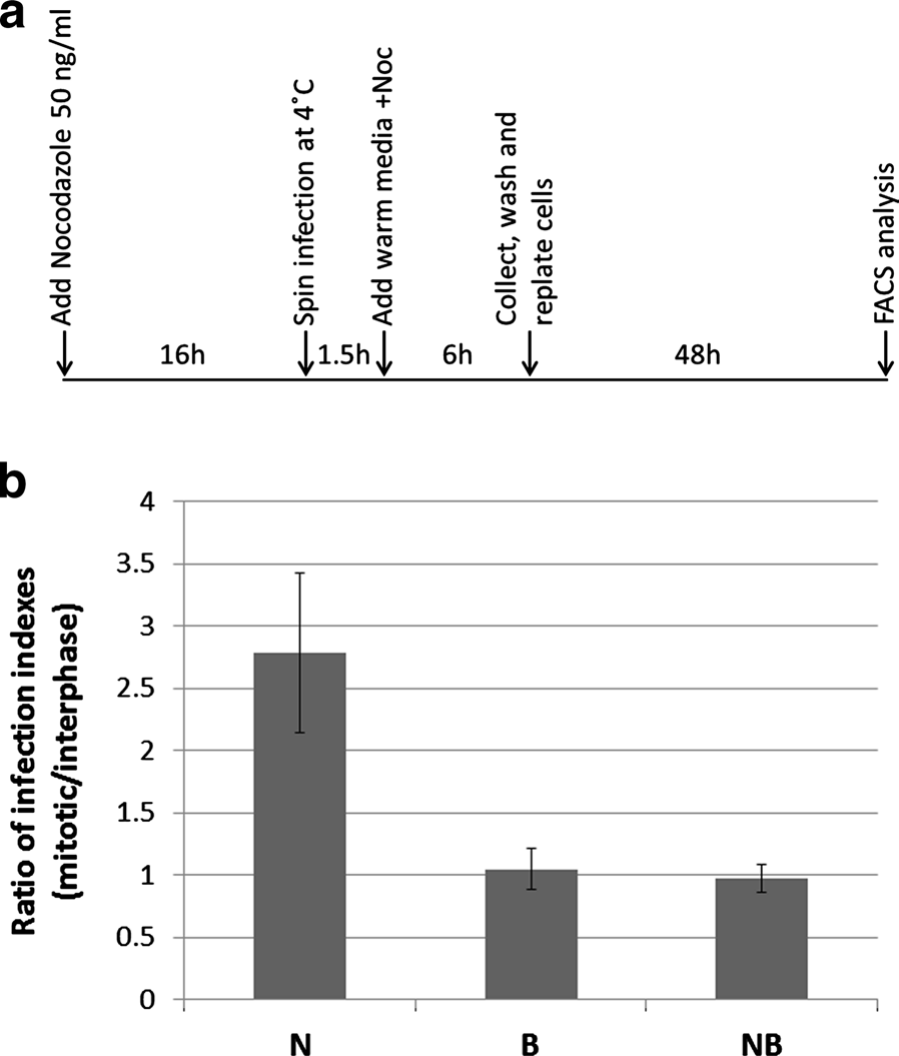
TRIM5α restriction is reduced in mitotic cells. **a** Timeline of nocodazole treatment and MLV infection of U/R cells. **b** Values of infection index (Methods) in nocodazole-arrested (mitotic) cells divided by infection index in interphase cells, for the N, B and NB -tropic viruses, harboring the pQCXIP-GFP-C1 vector. Bars represent the mean ± standard error of the means (n ≥ 3)

Tripartite motif 5 alpha (TRIM5α) is a host restriction factor that blocks infection of specific retroviruses in a species-dependent manner. Human TRIM5α restricts N-tropic MLV (N-MLV), but neither B-tropic MLV (B-MLV) nor the NB-tropic Moloney MLV [43]. TRIM5α is localized in ‘cytoplasmic bodies’, and these clusters are highly mobile, with long-distance movements along microtubules [44]. Recently, the importance of microtubule dynamics to TRIM5α function has been demonstrated as restriction to viral infection was reduced in unsynchronized cells treated with nocodazole or paclitaxel [22]. Since the microtubule network breaks down as the cell enters mitosis, a reduction in TRIM5α restriction may occur during this stage. To examine this, we first replaced portion of Moloney MLV Gag-Pol sequence with the cognate sequences derived from N or B -tropic clones (Methods). The amounts of N and B -tropic viruses, encapsidating the pQCXIP-GFP-C1 vector (Methods), were normalized by an exogenous RT assay and used to infect (M.O.I = 0.3) nocodazole-arrested or unsynchronized (untreated) U/R cells. Nocodazole was then removed, allowing the cells to cycle (see detailed timeline in Fig. 5a). Infection index was calculated as the percentage of GFP-positive cells multiplied by the geometric mean of the normalized GFP intensity in this sub-population, as determined by FACS analyses (Methods). In these experiments mitotic cells showed lower restriction for N-MLV compared to interphase cells, as revealed by a 2.7 fold higher infection index in mitotic cells (Fig. 5b). These results were in sharp contrast to those observed for B-MLV or NB-Moloney MLV (unrestricted strains), where no significant differences were observed for their infection index in mitotic versus interphase cells (Fig. 5b). This implies that in mitotic cells, TRIM5α restriction is reduced.

Taken together our results show that MLV is capable of fulfilling all early steps of infection in mitotic cells where no radial microtubules network is present. Moreover, the mitotic cell presents a reduced barrier towards restriction-sensitive MLV strains, suggesting that viral restriction may be sensitive to cell-cycle dependent alterations.

## Discussion

Due to the proliferative nature of the hematopoietic cells, the natural milieu encountered by MLV is expected to consist of cells at different stages of the cell cycle, including mitotic cells. Indeed, the process by which MLV gains access to the chromosomes involves the mitotic breakdown of the NE ([14] and recently visualized by us in live cells [15]). Whereas MLV infection was extensively studied in unsynchronized cells, the question whether MLV infection can occur ab initio in mitotic cells has yet to be addressed.

Endocytic entry is considered as a necessary step for the productive infection of ectropic MLV, due to the requirements for low pH [24–26] and cleavage by cathepsins` for envelope-mediated fusion [27–29]. However, among the different alterations to the cellular context, the mitotic cell is characterized by alterations to the organization and dynamics of membrane compartments including changes to the endocytic machinery [19, 31, 32]. Thus, concerning early steps of infection, we asked two basic questions: (1) does ectropic MLV enter mitotic cells? (2) does MLV entry involve arrival to internal membranous compartments in cells arrested at mitosis? The presence of viral particles inside the plasma membrane perimeter observed in confocal mid-planes of mitotic cells; and the engulfment of these particles by membranous compartments of the infected cells (Fig. 2) allowed us to conclude that the mitotic cell is permissive for ecotropic MLV entry. Thus, also in mitotic cells MLV reaches the intracellular compartments that supply the molecular requirements for fusion.

Upon exit from these compartments, reverse transcription should occur. We directly tested if the mitotic cellular milieu supports this process. Indeed, generation of gDNA could be readily detected in infected cells, arrested at mitosis (Fig. 4). Thus, all cellular requirements for reverse transcription, such as sufficient concentration of dNTPs, are met by the mitotic cell.

In interphase cells, the movement of virus-containing endosomes along polymerized microtubules may serve as a mechanism to enhance the efficiency of approaching the NE. Indeed, such proximity between viral cores and the NE allows the coincidence of nuclear entry by the cores and the initial stages of NE breakdown [15]. The usage of microtubules and related motor proteins for core trafficking and uncoating has been suggested also for HIV-1 [1–4]. Here we show that in mitotic cells, which are devoid of both intact NE and radial microtubule network, targeting of the mitotic chromosomes by the incoming viral cores still occurs, despite the lack of paths characteristic of active transport (Additional file 5: Fig. S4). Moreover, this process is relatively fast as cores that are docked to the chromosomes can be identified as early as 40 min postinfection (Figs. 2, 3). This implies that the microtubule network and the directed movement that it supports are not absolutely required for targeting the chromosomes. Moreover, the attachment to the chromosomes was followed by all subsequent steps of productive infection (measured upon reversal of the cell cycle arrest), since expression of GFP from MLV vector, which requires integration and transcription, was readily detected in cells where infection was initiated during mitosis.

The absence of a network of polymerized microtubules in mitotic cells, and the reported dependence on such network for optimal restriction of HIV infection by TRIM5α [22], raise the possibility that a similar scenario could occur in the context of N-tropic MLV infection of mitotic human cells. Thus, it is expected that in such cells, devoid of microtubule network, TRIM5α restriction should be specifically reduced towards the restricted MLV strain (N-tropic). Our results support this notion as GFP signals originating from N, but not B or NB, -tropic viruses were enhanced when infection was initiated in mitotic cells as compared to interphase cells. While changes to the cytoskeleton organization are a prominent feature of the altered cellular context of mitosis, additional changes in the milieu of mitotic cells may contribute to the reduced restriction.

## Conclusions

Altogether, MLV can infect interphase or mitotic cells with comparable efficiencies. Yet, this occurs through compound alterations to different parameters of MLV infection, imposed by the specific stage of the cell cycle. Whereas entry in interphase cells is more efficient, compared to mitotic cells, so is the TRIM5α-mediated restriction. Analogously, the barrier of the NE on core access to chromosomes, which exists in interphase cells, is absent in mitotic cells. These compensations result in similar infection outcomes. One can speculate that the ability to overcome restrictions in different cell contexts may contribute to viral diversity.

## Methods

### Cells

NIH3T3, 293T, U20S, U/R, U/R/H2A-RFP and U/R/EMTB-mCherry cell lines were grown as described before [15, 37]. The U/R/EMTB-mCherry cell line was generated by co-transfecting U/R cells (U2OS human osteosarcoma cells that express the MLV receptor [37]) with a plasmid expressing two mCherry repeats fused to the N-terminus of the microtubule binding-domain of ensconsin (EMTB [38]); and a plasmid expressing the puromycin-resistance gene (PAC). A colony stably expressing the EMTB-mCherry fusion protein was selected with puromycin (1 µg/ml)-containing media. To label U/R cells with the lipophilic dye DiD (1,1′-dioctadecyl-3,3,3′,3′-tetramethylindodicarbocyanine perchlorate; Life Technologies, V-22887), cells (~50 % confluency; 60 mm plate) were incubated with 5 µM DiD for 20 min in Opti-MEM medium (Life Technologies); after which, the cells were trypsinized, washed and replated in complete medium [Dulbecco’s Modified Eagle’s Medium (DMEM; Gibco) supplemented with 10 % fetal calf serum] with or without nocodazole. Concentrations of nocodazole (Sigma M1404) or 2-methoxyestradiol (2ME2; Sigma M6383) were 50 ng/ml and 1.3 mg/ml, respectively for all experiments. To enrich mitotic cells to about 90 %, rounded cells were detached from nocodazole treated-cultures by vigorously slapping the dishes (the mitotic shakeoff method; [14]) and equal number of floating cells were re-plated and incubated with the indicated virus.

To label infected U/R cells with both Hoechst 33342 dye and fluorescently labeled transferrin, unsynchronized cells were infected with DiD-labeled wt GFP virions (see below). At 1 h postinfection, Hoechst 33342 (Invitrogen; 0.02 mg/ml) and fluorescently labeled transferrin (Alexa 546-conjugated transferrin, Molecular Probes; 50 µg/ml) were added to the culture medium and interphase cells were imaged by time-lapse microscopy for up to 1 h.

To generate cells stably expressing fluorescently tagged mCAT-1, we co-transfected the plasmids mCAT-1–mStrawberry (10 µg) [41] and PGK-puro (1 µg, expressing the puromycin-resistance gene) into U2OS cells, using the PolyJet reagent (SignaGen Laboratories) according to the manufacture’s protocol. A colony, stably expressing the mStrawberry-tagged mCAT-1, was selected with puromycin (1 µg/ml)-containing media. Cells, expanded from this colony, were arrested (or not) with 2ME2 for 48 h, fixed with 4 % paraformaldehyde, stained with DAPI and visualized by confocal microscopy. Specifically, entire cell volumes were acquired under identical illumination conditions for all cells (nine cells for each experimental condition). Plasma membrane and adjacent intracellular regions were demarked by masking with Slidebook program and employed for calculation of mean intensity of fluorescence signal. Significance was calculated by Student’s *t* test.

### Viruses

The generation of MLV virions labeled with GFP-p12 fusion molecules (wt GFP virions) was as described before [15]. To label wt GFP virions with DiD, 293T cells that were transfected with plasmids expressing the components of wt GFP [15] were incubated 24 h posttransfection with 5 µM DiD for 4 h in Opti-MEM medium, after which the cells were washed and incubated with complete medium for additional 24 h. Virions-containing supernatants were filtered (0.45 µ), and frozen (−80 °C) in aliquots until use.

To examine the infectivity of the DiD-labeled wt GFP virions (GFP^+^DiD^+^), 293T cells that were transfected with plasmids expressing the components of wt GFP [15], in addition to a plasmid expressing the MLV-based vector pQCXIP-GFP-C1 [36], were divided 5 h posttransfection. One half was labeled with DiD as described above and the second half was left unlabeled. After additional 24 h, the culture supernatants were harvested and used to infect naïve U/R cells. 48 h postinfection, the numbers of GFP-positive cells and their mean fluorescence were quantified by FACS analyses.

wt Moloney MLV was harvested from cultures of chronically infected NIH3T3 cells. To encapsidate the pQCXIP-GFP-C1 vector [36] in wt Moloney MLV particles (NB-tropic), 293T cells (80 % confluency in a 60 mm plate) were co-transfected with pNCS plasmid (10 µg), expressing the wt Moloney MLV and the pQCXIP-GFP-C1 vector (5 µg). 48 h posttransfection the virion-containing culture supernatant was harvested, complemented with Hepes (50 mM; pH 7.4), filtered (0.45 µ) and kept frozen until use. To generate cognate particles with N- or B- tropism, a portion of Moloney MLV Gag-Pol sequence in pNCS was replaced with the related sequences derived from N or B -tropic MLV clones (pCIG3N or pCIG3B, generously provided by G. Towers, UCL), which contain the residues in capsid that define N and B tropism [45]. The resulting N or B -tropic clones were able to spread in NIH (which restrict B-tropic viruses) or U/R (which restrict N-tropic viruses) cells, respectively, with the same kinetics of the NB -tropic Moloney MLV (data not shown), demonstrating the expected tropism. These clones were co-transfected with pQCXIP-GFP-C1 vector, as above. Normalization of MLV virions was achieved by exogenous RT assay [46].

### Microscopy

Live-cell microscopy was performed essentially as described in [15]. For analyses, confocal movies were first deconvolved with the No Neighbours deconvolution algorithm of Slidebook software (Intelligent Imaging Innovations). Where indicated, time-composite channels were produced by Slidebook. Following this, GFP and/or DiD fluorescence were identified through intensity-based segmentation; the signal area, number of objects or overlap between objects were calculated by Slidebook. Path tracking was carried out with the particle tracking algorithm of SlideBook. Deconvolved movies were filtered with the Laplacian 2D filter of the same software. Objects in the filtered images were identified through intensity-based segmentation. Paths consisting of a minimum of 10 consecutive steps were approved by visual inspection before analysis. Maximal velocity values were determined for two groups of 19 paths by SlideBook. This software was also used to generate path coordinates for representative tracks, which were then used to calculate the cognate mean square displacement (MSD) values.

### Pcr

Low molecular DNA, containing the MLV genome and mitochondrial DNA, was extracted from infected cells at the indicated time points by the Hirt extraction method [42]. PCR amplification was applied using MLV specific primers (‘pNCS BsrGI FW’ 5′CCCAGGTTAAGATCAAGG3′ and ‘pNCS XhoI REV’ 5′CTTGGCCAAATTGGTGGG3′) and mitochondrial (cytochrome B-derived [47]; ‘CytB H15149′ 5′AAGCTTCCATCCAACATCTCAGCATGATGAAA3′ and ‘CytB L14841′ 5′ACTGCAGCCCCTCAGAATGATATTTGTCCTCA3′).

### Quantification of viral restriction

U/R cells in 6-well plates (~50 % confluence) were treated, or not, for 16 h with nocodazole. Subsequently, cells were infected (in the presence, or not, of nocodaxzole), for 6 h (M.O.I = 0.3), after which the cells were trypsinized, washed and replated with complete medium (with no nocodazole). Two days later, the cells were analyzed by FACS and infection index was calculated by multiplying the percentage of GFP-positive cells and the normalized geometric mean of the GFP signal [48]. Normalization took into account the increase in auto-fluorescence observed for all nocodazole-treated cells. For this, GFP signals in nocodazole-treated samples were corrected by division with the ratio of nocodazole-treated/untreated non-specific fluorescence values (~1.5).

## Additional files

10.1186/s12977-015-0220-2 Intracellular trafficking of MLV cores in interphase cells at 75 min postinfection. Unsynchronized U/R/EMTB-mCherry cells were infected with DiD-labeled wt GFP virions and interphase cells were imaged at 75 min postinfection. Time composites are shown in Fig. 1C. Viral cores are in green; DiD signal and EMTB-marked microtubules are pseudocolored in red and blue, respectively. Acquisition frequency: 0.4 frames per second (fps). Scale bar, 10 µm.
10.1186/s12977-015-0220-2 Movement of a MLV particle along a microtubule filament. A magnified view (from Additional file 1: Mov. S1) of a single GFP^+^DiD^+^ particle (yellow) that moves along a microtubule (pseudocolored blue). Acquisition frequency: 0.4 fps. Scale bar, 1 µm.
10.1186/s12977-015-0220-2 Movement of marked endosomes. U/R cells were transfected with plasmid expressing the endosome marker FYVE-GFP (green) and visualized by time-lapse microscopy two days posttransfection. Acquisition frequency: 1 fps. Scale bar, 10 µm.
10.1186/s12977-015-0220-2 Marked endosomes show two major types of movement. A magnified view (from Additional file 3: Mov. S3) of FYVE-GFP endosomes. Note the two largest endosomes that show a restricted movement relative to the other smaller endosomes, showing a directed movement. Acquisition frequency: 1 fps. Scale bar, 1 µm.
10.1186/s12977-015-0220-2 Endosomal localization of GFP^+^DiD^+^ particles. Unsynchronized U/R cells were infected with DiD-labeled wt GFP virions. At 1h postinfection, Hoechst 33342 dye and Alexa 546-conjugated transferrin were added to the culture and interphase cells were imaged by time-lapse microscopy (Methods). Inset marks the boundaries of Additional file 6: Mov. S5 and its separated channels are shown on the right. Viral cores are in green; DiD signal, transferrin and Hoechst 33342 are pseudocolored in red, blue and white, respectively. Scale bar, 10 µm.
10.1186/s12977-015-0220-2 Endosomal localization of GFP^+^DiD^+^ particles. Details are as in the legend of Additional file 5: Fig. S1. Triple co-localization of the three signals (blue-transferrin, red-viral membranes, and green viral-cores) is shown in white. Acquisition frequency: 0.13 fps.
10.1186/s12977-015-0220-2 Intracellular trafficking of MLV cores in interphase cells 24 h postinfection. U/R/EMTB-mCherry cells were infected with DiD-labeled wt GFP virions and interphase cells (same as in Fig. 1D) were imaged by time-lapse microscopy at 24 h postinfection. Top row, first frame of time-lapse. Bottom row, time composites. Viral cores are in green; DiD signal and EMTB-marked microtubules are pseudocolored in red and blue, respectively.
10.1186/s12977-015-0220-2 Intracellular trafficking of MLV cores in interphase cells at 24 h postinfection. Unsynchronized U/R/EMTB-mCherry cells were infected with DiD-labeled wt GFP virions and interphase cells were imaged at 24 h postinfection. Time composites are shown in Additional file 7: Fig. S2. Viral cores are in green; DiD signal and EMTB-marked microtubules are pseudocolored in red and blue, respectively. Acquisition frequency: 0.4 fps. Scale bar, 10 µm.
10.1186/s12977-015-0220-2 mCAT-1–mStrawberry localizes to the plasma membrane of mitotic and interphase cells. U2OS cells stably expressing mCAT-1–mStrawberry were arrested (B and D) or not (A and C) in mitosis with 2ME2. Cells were fixed, stained with DAPI, mounted and the entire cell volume was imaged by confocal microscopy. A and B are X, Z projections of an interphase cell (A) or a mitotic cell (B). C and D are single confocal planes of the cells depicted in A and B. mCAT-1–mStrawberry is shown in red, DAPI in blue. Bars, 10 µm.
10.1186/s12977-015-0220-2 Path analysis of MLV cores in interphase and mitotic cells. U/R/EMTB-mCherry cells in interphase, or arrested at mitosis, were infected with wt GFP virions and imaged by time-lapse microscopy at 75 min postinfection. Paths of intracellular cores were analyzed by SlideBook software. (A) Distribution of frequencies of maximal velocity values of cores in mitotic (dark gray) and interphase (light gray) cells are depicted (as percentage of cores with the indicated maximal velocity). (B) and (C) MSD (pixels^2^) plots against time in seconds (s) of representative paths in interphase (B) and mitotic (C) cells. Pixel size was 160 nm. Graphs showing linear, exponential-like and unclassified MSD/time ratios are labeled in blue, red and gray colors, respectively.
10.1186/s12977-015-0220-2 Movement of MLV core confined by endosome borders. U/R/H2A-RFP cells with DiD-labeled cellular membranes were arrested with nocodazole, infected with wt GFP and visualized 1 h postinfection, as in Fig. 2B. Shown a magnification of a confocal, intracellular mid-plane with a DiD-labeled endosome (pseudocolored red) that engulfs one to two labeled MLV cores (green). Fluorescent signals are presented as time composites. A restricted movement of another core, attached to the chromosome (pseudocolored blue), is shown as a reference. Acquisition frequency: 0.8 fps. Scale bar, 1 µm.
10.1186/s12977-015-0220-2 MLV core docking to mitotic chromosomes in absence of microtubules in U/R/EMTB-mCherry cells. 2ME2-treated U/R/EMTB-mCherry cultures were infected with wt GFP and imaged by time-lapse microscopy at 45 min postinfection. EMTB-mCherry and viral cores are in red and green, respectively; condensed chromosomes appear as intracellular dark regions. A kymograph of this movie is presented in Fig. 3B. Acquisition frequency: 1.2 fps. Scale bar, 10 µm.
10.1186/s12977-015-0220-2 Docking of MLV cores to mitotic chromosomes in absence of microtubules in NIH3T3/EMTB-mCherry cells. Nocodazole-treated NIH3T3/EMTB-mCherry cultures were infected with wt GFP and imaged by time-lapse microscopy at ~2 h postinfection, after staining the chromosomes with DAPI. EMTB-mCherry, viral cores and mitotic chromosomes are in red, green and blue, respectively. Acquisition frequency: 0.1 fps. Scale bar, 10 µm.
