# Supplementary figures and images for: Dynamics and restriction of murine leukemia virus cores in mitotic and interphase cells
Source: Retrovirology. 2015 Nov 14;12:95. doi: 10.1186/s12977-015-0220-2 (PMC4650138; doi:10.1186/s12977-015-0220-2)

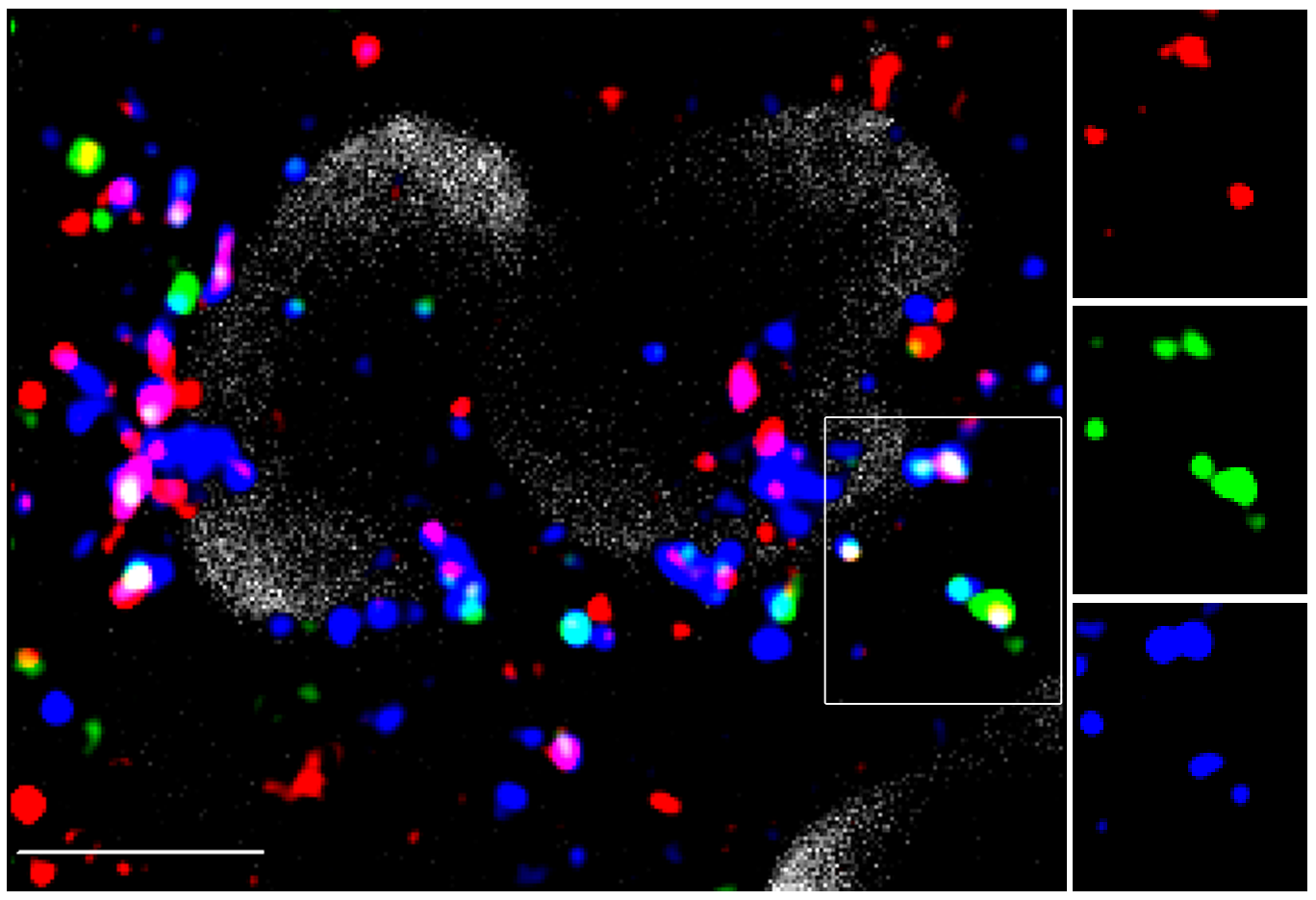

Supplement: Supplementary file 5 — 10.1186/s12977-015-0220-2 Endosomal localization of GFP+DiD+ particles. Unsynchronized U/R cells were infected with DiD-labeled wt GFP virions. At 1h postinfection, Hoechst 33342 dye and Alexa 546-conjugated transferrin were added to the culture and interphase cells were imaged by time-lapse microscopy (Methods). Inset marks the boundaries of Additional file 6: Mov. S5 and its separated channels are shown on the right. Viral cores are in green; DiD signal, transferrin and Hoechst 33342 are pseudocolored in red, blue and white, respectively. Scale bar, 10 µm. [file 12977_2015_220_MOESM5_ESM.tif]

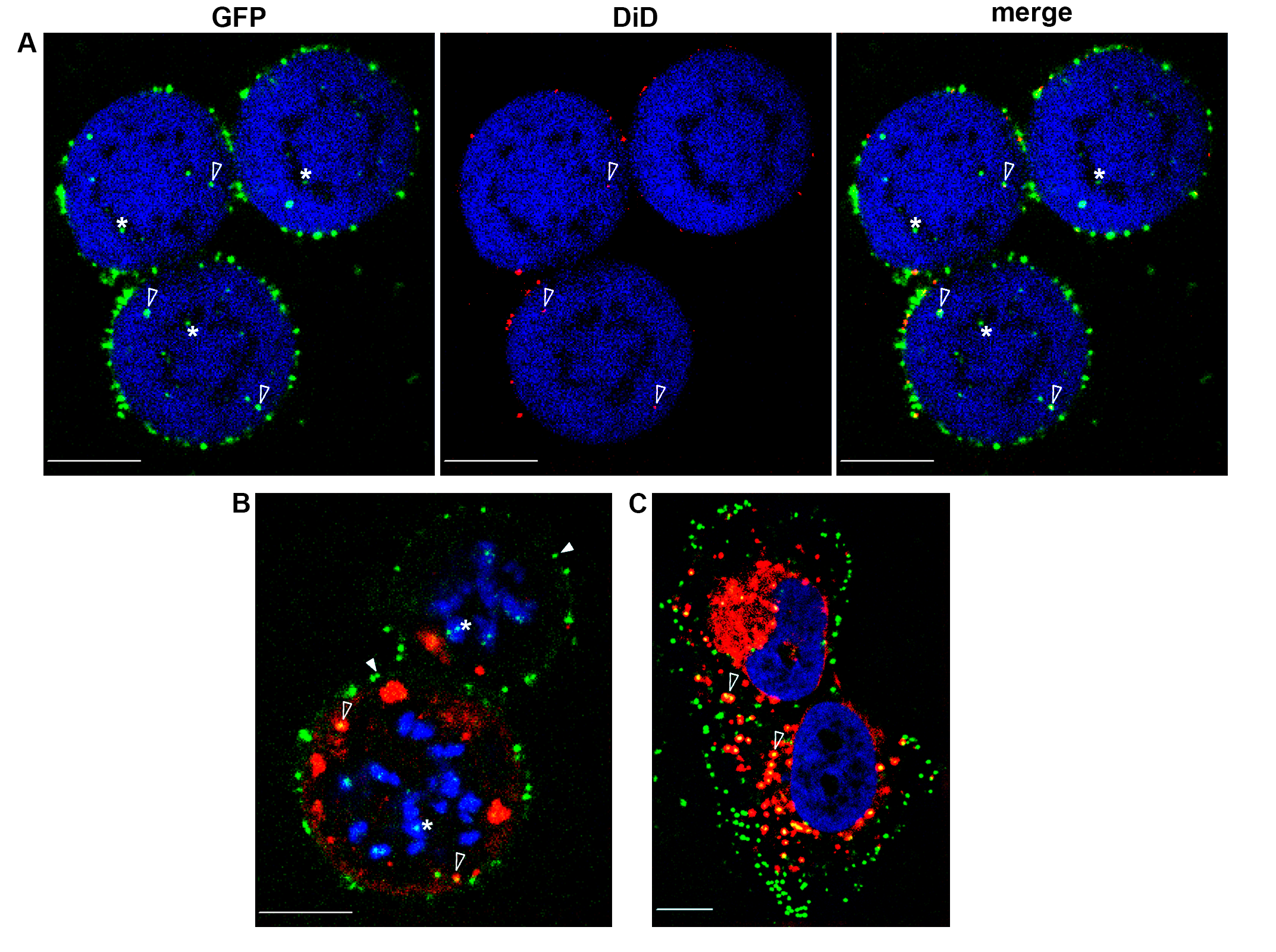

Supplement: Supplementary file 7 — 10.1186/s12977-015-0220-2 Intracellular trafficking of MLV cores in interphase cells 24 h postinfection. U/R/EMTB-mCherry cells were infected with DiD-labeled wt GFP virions and interphase cells (same as in Fig. 1D) were imaged by time-lapse microscopy at 24 h postinfection. Top row, first frame of time-lapse. Bottom row, time composites. Viral cores are in green; DiD signal and EMTB-marked microtubules are pseudocolored in red and blue, respectively. [file 12977_2015_220_MOESM7_ESM.tif]

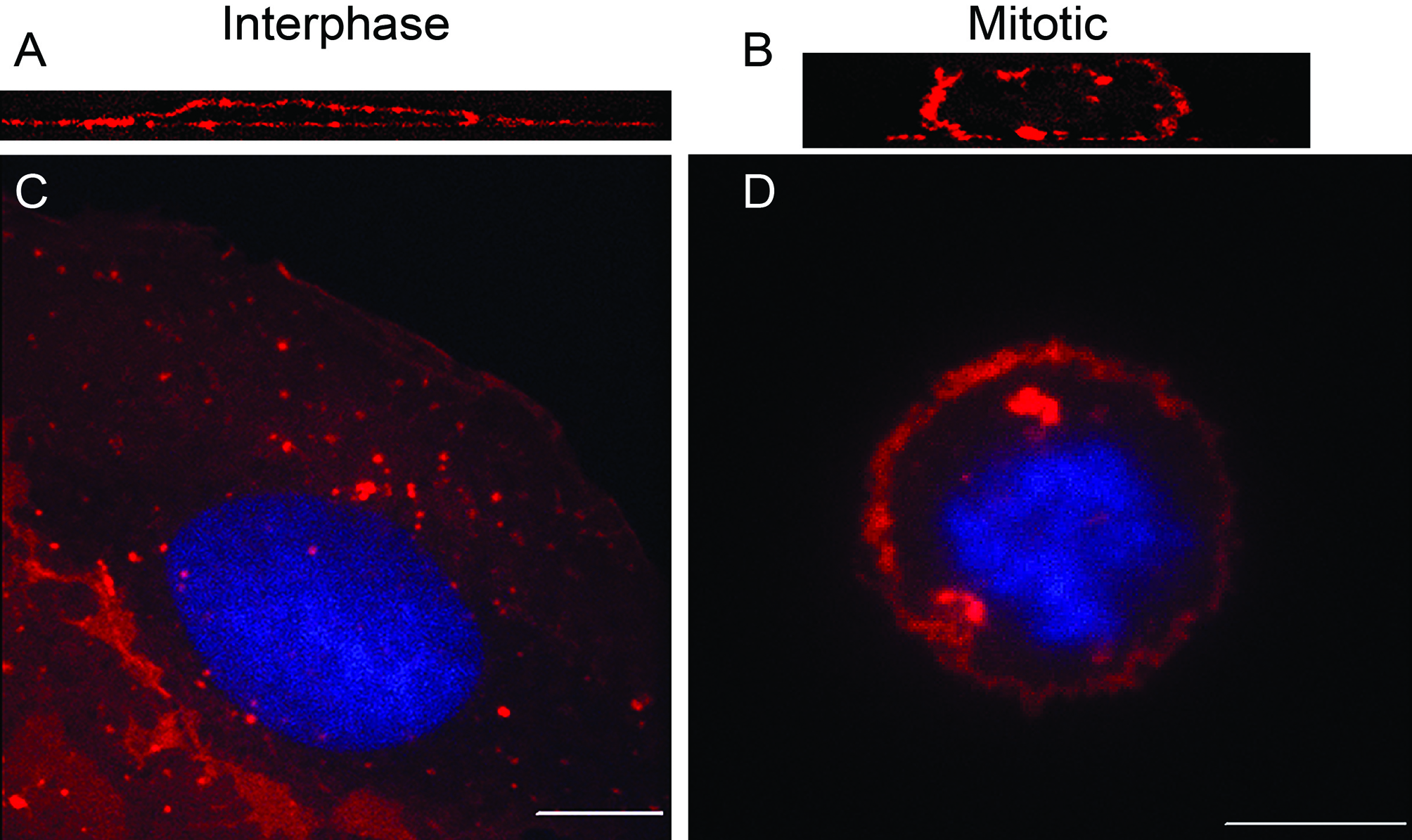

Supplement: Supplementary file 9 — 10.1186/s12977-015-0220-2 mCAT-1–mStrawberry localizes to the plasma membrane of mitotic and interphase cells. U2OS cells stably expressing mCAT-1–mStrawberry were arrested (B and D) or not (A and C) in mitosis with 2ME2. Cells were fixed, stained with DAPI, mounted and the entire cell volume was imaged by confocal microscopy. A and B are X, Z projections of an interphase cell (A) or a mitotic cell (B). C and D are single confocal planes of the cells depicted in A and B. mCAT-1–mStrawberry is shown in red, DAPI in blue. Bars, 10 µm. [file 12977_2015_220_MOESM9_ESM.tif]

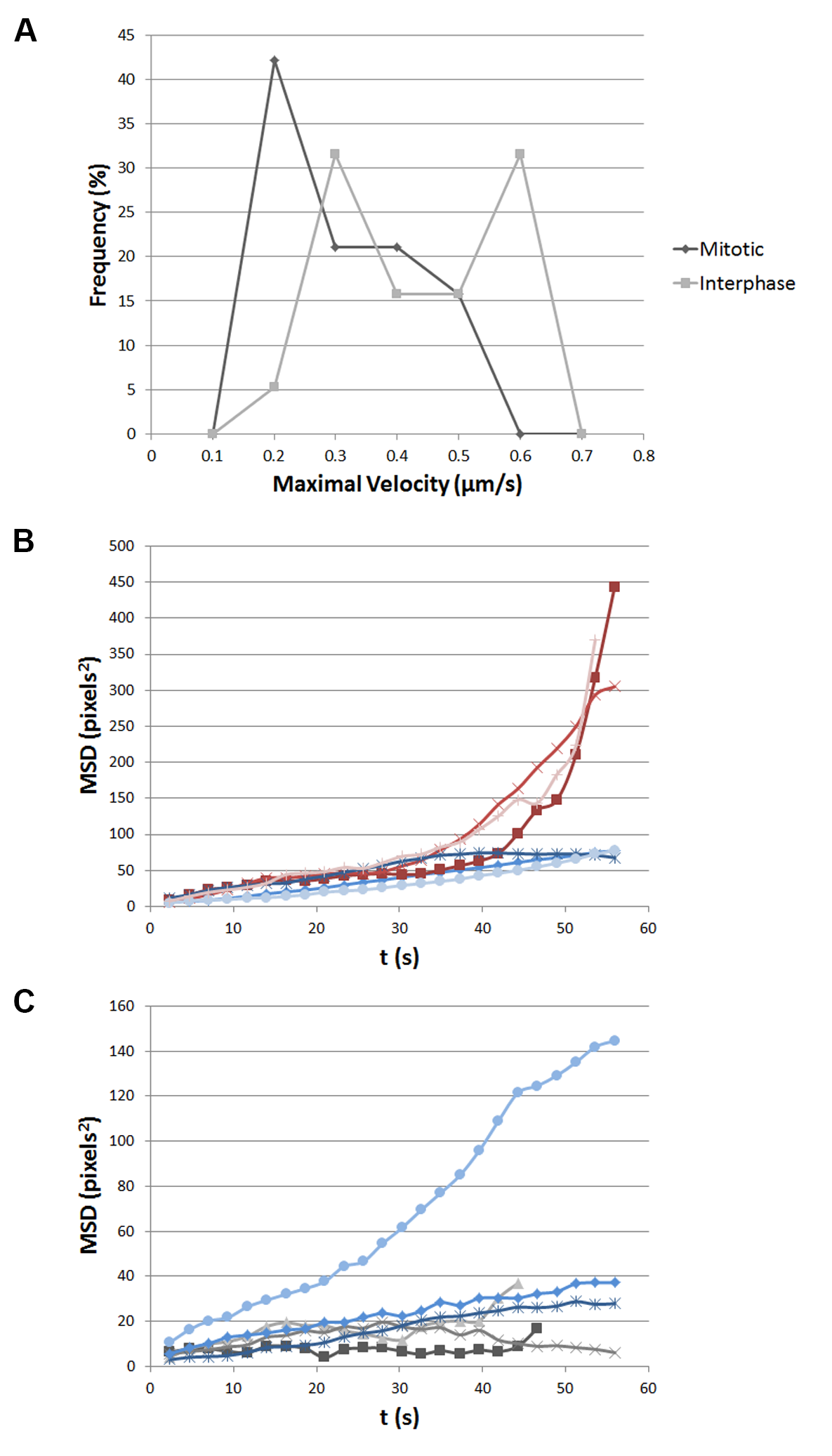

Supplement: Supplementary file 10 — 10.1186/s12977-015-0220-2 Path analysis of MLV cores in interphase and mitotic cells. U/R/EMTB-mCherry cells in interphase, or arrested at mitosis, were infected with wt GFP virions and imaged by time-lapse microscopy at 75 min postinfection. Paths of intracellular cores were analyzed by SlideBook software. (A) Distribution of frequencies of maximal velocity values of cores in mitotic (dark gray) and interphase (light gray) cells are depicted (as percentage of cores with the indicated maximal velocity). (B) and (C) MSD (pixels2) plots against time in seconds (s) of representative paths in interphase (B) and mitotic (C) cells. Pixel size was 160 nm. Graphs showing linear, exponential-like and unclassified MSD/time ratios are labeled in blue, red and gray colors, respectively. [file 12977_2015_220_MOESM10_ESM.tif]
